# Solvent-Dependent Monomer Leaching From Two Commercial Dental Resin Composites: A Time-Dependent Elution Behaviour

**DOI:** 10.7759/cureus.117193

**Published:** 2026-09-30

**Authors:** Vidushi Gilhotra, Rahul Banerjee, Ajay Chhabra, Shayan Karak, Supratim Ghosh, Bhupendra Ramesh Babaria

**Affiliations:** 1 Dentistry, All India Institute of Medical Sciences, Kalyani, Kalyani, IND; 2 Chemistry, Indian Institute of Science Education and Research Kolkata, Kalyani, IND; 3 Conservative Dentistry and Endodontics, All India Institute of Medical Sciences, Kalyani, Kalyani, IND

**Keywords:** biocompatibility, dental resin composite, elution, high-resolution mass spectrometry, residual monomer, tegdma, ultraviolet–visible spectroscopy

## Abstract

Background: Resin-based composites remain incompletely polymerised after light-curing, leaving unreacted monomers that can leach into oral fluids. Data on the temporal patterns of this release - especially contrasting flowable and packable composites under varying solvent conditions - remain limited.

Materials and methods: This in vitro study evaluated the identity and time-dependent elution of residual monomers from a packable composite (Charisma Smart) and a flowable composite (Fusion Flo). Specimens (n = 10 per composite × medium combination; 40 total) were immersed continuously in either deionised water or a 1:1 water-ethanol mixture. The extraction medium was fully replenished on Days 1, 2, 7, and 14, tracking the same specimens longitudinally (160 eluates total). High-resolution mass spectrometry (HRMS) qualitatively identified leached species. Ultraviolet-visible (UV-Vis) spectrophotometry tracked relative monomer quantities calibrated against a triethylene glycol dimethacrylate (TEGDMA) standard; for co-eluting species, values are reported as approximate methacrylate equivalents. Data were analysed using a split-plot three-way analysis of variance (ANOVA) with Tukey's HSD post-hoc tests. Qualitative scanning electron microscopy (SEM) surveyed surface topography before and after 30-day immersion.

Results: Monomer leaching differed significantly by composite, medium, interval, and all interactions (p < 0.001). Water-ethanol produced substantially greater leaching than water for both composites (p < 0.05), with the packable composite releasing the largest absolute quantities (1,086.1 µg at Day 1). HRMS also detected urethane dimethacrylate (UDMA) exclusively in the flowable composite's water-ethanol eluate. Only the flowable composite in water exhibited a simple monotonic decline across intervals. The other three conditions showed a sharp initial drop from Day 1 to Day 2, followed by a significant secondary rise at Day 7. SEM confirmed structural surface cracking and flaking in both media, alongside filler-scale pitting on water-ethanol-immersed surfaces.

Conclusion: Both composites released statistically distinguishable amounts of monomer, heavily accelerated by the water-ethanol medium. The non-monotonic temporal pattern reveals a complex secondary release phase not captured by standard single-decay models, highlighting the critical relevance of observation duration and solvent dynamics in biocompatibility screenings.

## Introduction

Resin-based composites are now the restorative material of choice in contemporary operative dentistry, valued for their aesthetics, conservative cavity design, and direct chairside placement [1]. Their organic matrix is typically built from dimethacrylate monomers - bisphenol A glycidyl methacrylate (Bis-GMA), urethane dimethacrylate (UDMA), ethoxylated bisphenol A dimethacrylate (Bis-EMA), and triethylene glycol dimethacrylate (TEGDMA), the last added as a diluent to reduce viscosity and permit adequate handling and filler loading [2,3]. Photopolymerisation of these matrices is never complete: reported degree-of-conversion values typically range from 40% to 75%, depending on monomer chemistry, filler content, and curing parameters, leaving a variable fraction of unreacted double bonds trapped in the cured network [2,4].

This residual, unpolymerised fraction is not chemically inert. Once a restoration is exposed to the oral cavity's aqueous, enzymatically active environment, water sorption swells the polymer network, hydrolyses ester linkages, and drives the progressive outward diffusion of unreacted monomers and low-molecular-weight degradation products into saliva, crevicular fluid, and, by extension, pulpal tissue [2,5]. A comprehensive meta-analysis of the dental materials literature confirmed that resin-based restoratives reliably release Bis-GMA, UDMA, TEGDMA, and Bis-GMA-derived bisphenol A (BPA) in analytically detectable quantities, with substantially greater release into organic or ethanol-containing media than into water alone [1].

Leaching of unreacted monomer is a clinically meaningful problem rather than an analytical curiosity. A dental restoration sits in continuous contact with the tooth and surrounding tissues for years, so even low concentrations of leachable monomer amount to chronic, low-dose exposure: residual monomer released at the restoration margin can diffuse through remaining dentine to reach the pulp, be taken up directly by gingival and mucosal tissue, or be swallowed in saliva and absorbed through the gastrointestinal tract, giving it reach well beyond the oral cavity. TEGDMA has drawn particular attention here. At concentrations comparable to those estimated at the pulp-dentine interface, it induces concentration- and time-dependent cytotoxicity in dental pulp cells through both caspase-dependent and caspase-independent apoptotic pathways, alongside oxidative DNA damage, cell-cycle arrest, and impaired mineralisation-related gene expression [6-10]. It has also been shown to cause DNA double-strand breaks in gingival fibroblasts adjacent to the restoration margin [11], to induce genotoxicity in macrophages via DNA fragmentation and caspase activation once it reaches the systemic circulation [9], and to provoke oxidative stress and inflammatory responses in oral epithelial cells, consistent with its recognised role as a contact allergen. Bis-GMA-related BPA raises a related, separate concern: several groups have documented transient elevations in salivary and urinary BPA after composite placement, though systemic exposure appears to remain below established toxicological thresholds [12,13]. Across the pulp, periodontium, immune cells, oral mucosa, and, through BPA, the endocrine system, this evidence base is why residual monomer leaching is treated as a genuine biocompatibility concern rather than a cosmetic or purely chemical one.

Comparatively few studies, however, have examined how the leaching profile evolves over sequential, medium-term extraction intervals in a design that directly contrasts a flowable and a packable composite - two categories that differ considerably in filler loading, viscosity, and clinical indication, and so might be expected to differ in release kinetics too [4,5]. While HPLC has long been the workhorse for quantifying eluted monomers, HRMS offers superior specificity for identifying leached species by exact mass, while UV-Vis spectrophotometry adds a rapid, low-cost way to track relative changes in leaching intensity over time [1,14].

The objective of this study was to characterise, using a combined HRMS and UV-Vis approach, both the chemical identity and the temporal elution pattern of residual methacrylate monomers released from a commercially available flowable composite (Fusion Flo) and a packable composite (Charisma Smart, Kulzer) into water and into a 1:1 water-ethanol medium over a 14-day observation period. It was hypothesised that (i) TEGDMA (relative release), owing to its relatively hydrophilic character and diluent role, would be the most consistently detected leachable species across both materials and media; (ii) the water-ethanol medium would elicit greater leaching than water alone, in keeping with the established solubility-parameter relationship between monomer hydrophobicity and extraction solvent polarity; and (iii) leaching intensity would decline progressively across successive collection intervals, reflecting depletion of the readily accessible surface and near-surface monomer fraction.

## Materials and methods

Study design and materials

This in vitro comparative elution study evaluated two commercially available light-cured resin composites representing distinct handling categories: a packable, hybrid-filler composite (Charisma Smart, Kulzer GmbH, Hanau, Germany) and a low-viscosity flowable composite (Fusion Flo, Prevest DenPro Limited, India). Based on the manufacturer-declared composition of both materials, four dimethacrylate monomers were identified a priori as the principal candidate leachable species: Bis-GMA (relative molecular mass 512.60), Bis-EMA (452.55), UDMA (470.56), and TEGDMA (286.32) (refer to Figure 1 for Schematics of the procedure).

**Figure 1:**
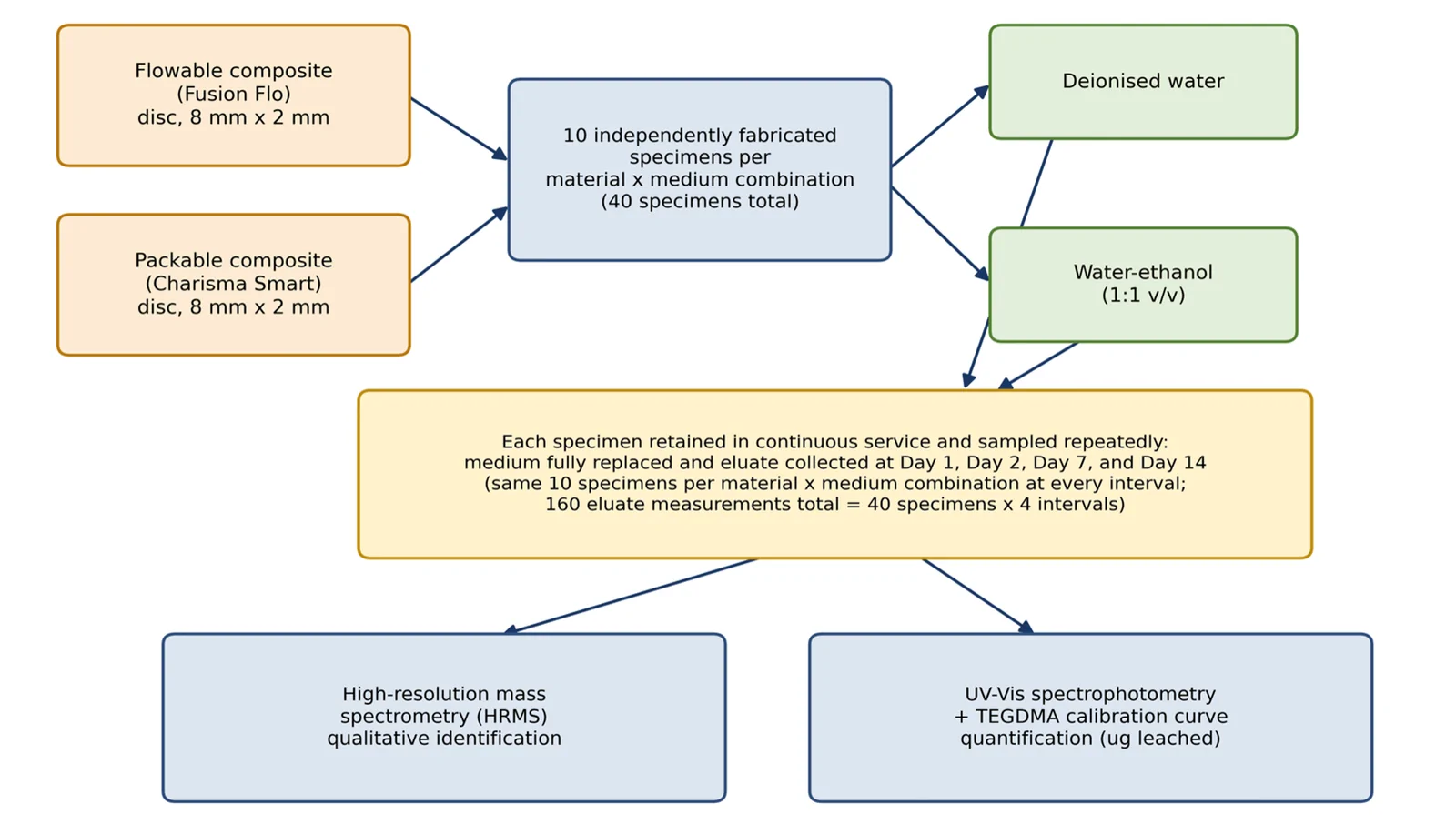
Schematic of the experimental design and analytical workflow. Ten independently fabricated specimens were assigned to each material × medium combination (40 specimens in total); each specimen was retained in continuous service and sampled repeatedly, with the extraction medium fully replaced on Days 1, 2, 7, and 14, generating 160 eluate measurements in total. Created by the author with Adobe Illustrator software (Adobe Inc., San Jose, CA)

Specimen preparation

Disc-shaped specimens (8 mm diameter × 2 mm thickness) were fabricated for each material using a Teflon mould. Ten independent specimens were prepared for each material × medium combination (40 specimens in total), each placed on a Mylar-covered glass slab to minimise the oxygen-inhibited surface layer, in accordance with standard protocols used in comparable elution studies [4,5]. Each specimen was light-cured from the top surface using a light-emitting diode (LED) curing unit operating in the 440-480 nm wavelength range at an irradiance of approximately 1,000-1,200 mW/cm², for 20 seconds, consistent with manufacturer instructions and with curing parameters commonly reported in the elution literature [4,5]. Irradiance was verified using a dental radiometer prior to specimen fabrication. Specimens were stored dry in the dark at room temperature for 24 hours after curing to allow the post-cure polymerisation reaction to approach completion before extraction commenced, mirroring the conditioning interval used in comparable HPLC-based elution protocols [4]. The Mylar cover limited oxygen inhibition at the top and bottom flat faces cast against it; the disc's cylindrical edge, formed against the Teflon mould wall rather than Mylar, was not specifically characterised for cure completeness and may retain a distinct, less well-polymerised surface layer relative to the flat faces.

Extraction protocol

Each specimen was placed in its own 2-mL borosilicate glass vial containing 1 mL of extraction medium: either freshly generated ASTM type I deionised water, or a 1:1 (v/v) deionised water-absolute ethanol mixture, the latter selected not as a physiological surrogate but as an accelerated, aggressive extraction model designed to establish the upper-bound ceiling of monomer release under stressed solvent conditions, deliberately exceeding the alcohol concentration and duration of typical dietary or oral-hygiene exposures [11]. Vials were maintained at 37 °C in the dark to simulate the intraoral thermal environment while minimising photodegradation of the eluate.

The dual mechanisms driving material degradation and enhanced monomer liberation within these media - specifically interfacial ester hydrolysis and solvent-driven network swelling - are illustrated schematically in Figure 2.

**Figure 2:**
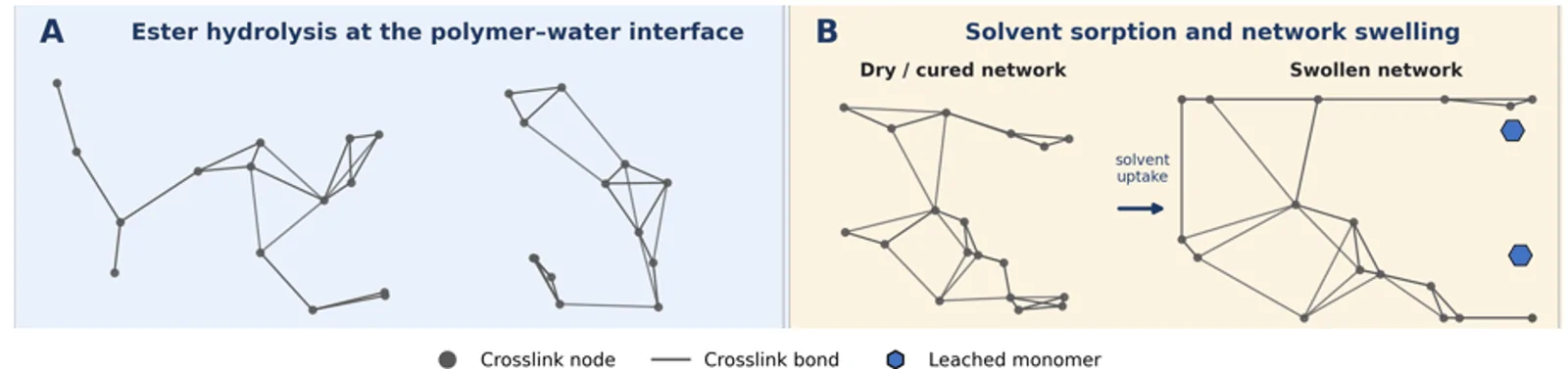
Proposed chemical and structural mechanisms governing monomer elution. (A) Hydrolytic cleavage at the polymer-water interface, where water molecules undergo nucleophilic attack on pendant ester crosslinks, releasing low-molecular-weight degradation fragments. (B) Solvent sorption and network swelling, demonstrating how water and ethanol diffuse into the cured dimethacrylate matrix, increasing crosslink mesh spacing (free volume) and creating expanded diffusion pathways for trapped residual monomers. Created by the author with Adobe Illustrator software (Adobe Inc., San Jose, CA)

Eluate was collected and the extraction medium fully replenished at four sequential intervals - Days 1, 2, 7, and 14 - with each specimen retained in continuous service throughout the 14-day protocol rather than being replaced with a fresh specimen at each interval; the same 10 specimens per material × medium combination therefore contributed a measurement at every one of the four intervals. Each collected eluate accordingly represented monomer release accruing during that specific inter-collection period rather than a cumulative concentration within a static solution, allowing the temporal pattern of release to be resolved for each individual specimen rather than obscured by progressive solvent saturation. Applied across two materials, two extraction media, and four collection intervals, with 10 replicate specimens per material × medium combination, this design generated a total of 160 eluate measurements (40 specimens, each sampled on four occasions) for analysis.

High-resolution mass spectrometric analysis

Each eluate sample was analysed directly by HRMS in positive-ion mode to identify relative release of leached monomer-related species according to their accurate mass-to-charge (m/z) ratios and characteristic adduct patterns (principally (M+H)⁺ and (M+Na)⁺). Candidate assignments were made by matching observed accurate masses, within a tolerance appropriate to the instrument's mass accuracy, against the theoretical exact masses of Bis-GMA, Bis-EMA, UDMA, and TEGDMA and their sodiated and protonated adducts. HRMS therefore served as the primary qualitative tool for confirming the chemical identity of the eluted species, complementing the quantitative and semi-quantitative information obtained from UV-Vis spectrophotometry.

Ultraviolet-visible spectrophotometric analysis

UV-Vis absorption spectra of all 160 eluates were recorded over the 200-400 nm range using a double-beam spectrophotometer, with the corresponding fresh, unexposed extraction medium used as the blank in each case. UV-Vis served here as a rapid screening and relative-comparison tool rather than a validated quantitative method for multi-monomer matrices. A calibration curve was constructed from serial dilutions of a relative release of TEGDMA reference standard in each corresponding extraction medium, and absorbance at the TEGDMA-associated wavelength was converted to a mass using this calibration relationship. Where HRMS confirmed only TEGDMA was present, this value is reported as TEGDMA leached (µg) per specimen. For the one condition where HRMS also detected co-eluted UDMA (see Results), this approach has a definitive limitation: because TEGDMA and UDMA absorb at overlapping low-UV wavelengths and have different molar absorptivities, single-wavelength UV-Vis cannot resolve their individual contributions to the measured signal, and the same calibration relationship therefore yields only an approximate relative release, TEGDMA-calibrated methacrylate-equivalent value rather than a chemically precise TEGDMA or total-methacrylate mass for that condition.

Scanning electron microscopy (SEM) surface analysis

To complement the chemical and spectroscopic elution data with direct morphological evidence of solvent-induced surface change, one flowable specimen (specimen A) and one packable specimen (specimen B) were examined by SEM, fabricated using the same protocol described above. For each material, a baseline specimen was imaged prior to any immersion; separate specimens were then imaged after 14 days of continuous immersion in deionised water or in the 1:1 water-ethanol medium. Specimens were imaged in secondary electron (SED) mode on a SEM (JEOL) operating at 5.00 kV accelerating voltage and a working distance of approximately 25-28 mm, at magnifications ranging from ×16 (whole-specimen overview; field of view approximately 6-8 mm) to ×65 (high-magnification surface detail; field of view approximately 1.5-2 mm), with acquisition parameters and a scale bar embedded in each micrograph. Magnification was not held constant across all specimens and media (see Results); this analysis therefore functions as a qualitative, descriptive survey of general surface topographic change rather than a magnification-matched comparative study, and no quantitative comparison of surface features between media is intended or supported by this method.

Statistical analysis

Data of all the samples were entered into Microsoft excel (Microsoft® Corp., Redmond, WA) and analyses using Stata Statistical Software Release 17 (StataCorp LLC, College Station, TX), since the same 10 specimens per composite × medium combination were sampled repeatedly at all four collection intervals, TEGDMA quantities (µg leached per specimen) were compared using a split-plot (repeated-measures) three-way analysis of variance (ANOVA), with composite (flowable, packable) and extraction medium (water, water-ethanol) treated as between-specimen factors and collection interval (Days 1, 2, 7, and 14) treated as a within-specimen factor. Between-specimen effects (composite, medium, and their interaction) were tested against the specimen-nested-within-condition error term; within-specimen effects (interval and its interactions with composite and medium) were tested against the interval × specimen residual error term. Because interval was a repeated-measures factor, sphericity was assessed using Mauchly's test; where violated, degrees of freedom for within-specimen effects were adjusted using the Greenhouse-Geisser correction (see Table 3 note). Post-hoc pairwise comparisons used Tukey's honestly significant difference (HSD) test, calculated separately for the between-specimen and within-specimen comparison families using their respective error terms. Statistical significance was set at α = 0.05. All 160 eluate measurements (40 specimens × 4 intervals) were included. HRMS species identification remained qualitative, as described above.

## Results

Chemical identity of leached species

HRMS analysis identified a consistent signal corresponding to TEGDMA, detected as its sodium adduct (M+Na)⁺ at m/z 309, in the eluates of both the flowable and the packable composite and in both extraction media, across all four collection intervals. This finding indicates that relative release of TEGDMA was the most readily and consistently detectable leachable monomer in the present system, irrespective of composite type or solvent polarity.

A material-specific difference emerged with respect to UDMA. In the flowable composite, an additional HRMS signal attributable to UDMA was detected exclusively in the water-ethanol medium and was not observed in the corresponding water-only eluates. No UDMA-associated signal was detected in either extraction medium for the packable composite. Neither Bis-GMA nor Bis-EMA produced a clearly assignable signal in any eluate under the conditions used in this qualitative HRMS analysis (Table 1). Therefore, their non-detection should be interpreted cautiously, as it may reflect either limited release from the cured matrix and/or concentrations below the detection capability of the present analytical method.

**Table 1:** Summary of HRMS-detected species and UV-Vis absorption maxima by composite and extraction medium (species identity, pooled across the four collection intervals). HRMS: High-resolution mass spectrometry; TEGDMA: Triethylene glycol dimethacrylate; UDMA: Urethane dimethacrylate

Composite	Extraction medium	HRMS-detected species	UV-Vis absorption maxima (nm)
Flowable (Fusion Flo)	Water	TEGDMA	214, 314 (lower intensity)
Flowable (Fusion Flo)	Water-ethanol (1:1)	TEGDMA, UDMA	214, 314 (higher intensity)
Packable (Charisma Smart)	Water	TEGDMA	214
Packable (Charisma Smart)	Water-ethanol (1:1)	TEGDMA	214, 280 (new peak)

Quantitative TEGDMA leaching

TEGDMA quantities leached (relative release) per specimen (mean ± SD, n = 10) across all 16 material × medium × interval combinations are summarised in Table 2 and Figure 3; for the flowable composite's water-ethanol condition specifically, HRMS also detected co-eluted UDMA (Table 1), and because the two monomers absorb in the same low-UV region and have different molar absorptivities, values reported for that one condition are an approximate TEGDMA-calibrated methacrylate-equivalent signal rather than a precise TEGDMA or total-methacrylate mass (see Table 2 note). The split-plot three-way ANOVA (Table 3) found significant between-specimen effects of composite and medium and their interaction, and significant within-specimen effects of collection interval and its interactions with composite and medium (all p < 0.001), indicating that the effect of medium on leaching depended on which composite and which timepoint were being compared, and vice versa.

**Table 2:** Descriptive statistics for TEGDMA leached (relative release) (µg per specimen, mean ± SD, n = 10 per cell). TEGDMA: triethylene glycol dimethacrylate

Composite	Medium	Day 1	Day 2	Day 7	Day 14
Flowable	Water	126.1 ± 4.2	43.5 ± 2.6	35.3 ± 2.1	10.6 ± 0.8
Flowable	Water-ethanol	471.5 ± 10.7	61.5 ± 2.3	116.7 ± 3.9	125.9 ± 2.4
Packable	Water	134.2 ± 4.3	20.5 ± 1.7	31.0 ± 2.4	7.6 ± 0.8
Packable	Water-ethanol	1086.1 ± 7.7	412.7 ± 3.4	933.7 ± 4.5	293.5 ± 2.5

**Table 3:** Split-plot three-way ANOVA summary (composite × medium × day). Material, medium, and material × medium are between-specimen effects, tested against the specimen-nested-within-condition error term (df = 36). ᴳᴳDay and its interactions with material and medium are within-specimen effects; sphericity was violated (Mauchly's W = 0.232, χ²(5) = 55.07, p < 0.001, ε = 0.539), so Greenhouse-Geisser-corrected degrees of freedom are reported for these rows (ε applied to the nominal df = 3, 108 error term) rather than the uncorrected values; this correction did not change which effects were significant. The F Column: The two distinct strings of numbers under the "F" column represent the calculated F-statistic values from the three-way ANOVA test. They measure the ratio of systematic variance (the effect of the variable) to random error variance. Specifically, 84,431, 217,223, and 88,359 correspond to the between-specimen effects (material, medium, and their interaction), tested using the error tracking of the specific fabricated specimens; 64,717, 13,561, 31,521, and 12,769 correspond to the within-specimen time-series factors (day and its multi-variable interactions). The GG superscript stands for the Greenhouse-Geisser correction. Because a single group of specimens was evaluated repeatedly across multiple time intervals, a standard variance rule called "sphericity" was violated during statistical testing. The Greenhouse-Geisser correction adjusts the degrees of freedom downward (from the standard whole numbers to decimals such as 1.62 and 58.25) to prevent false-positive errors, ensuring that the final 𝑝-value (𝑝 < 0.001) remains statistically reliable.

Source	df	df (error)	F	p
Material	1	36	84,431	<0.001
Medium	1	36	217,223	<0.001
Material × Medium	1	36	88,359	<0.001
Dayᴳᴳ	1.62	58.25	64,717	<0.001
Material × Dayᴳᴳ	1.62	58.25	13,561	<0.001
Medium × Dayᴳᴳ	1.62	58.25	31,521	<0.001
Material × Medium × Dayᴳᴳ	1.62	58.25	12,769	<0.001

**Figure 3:**
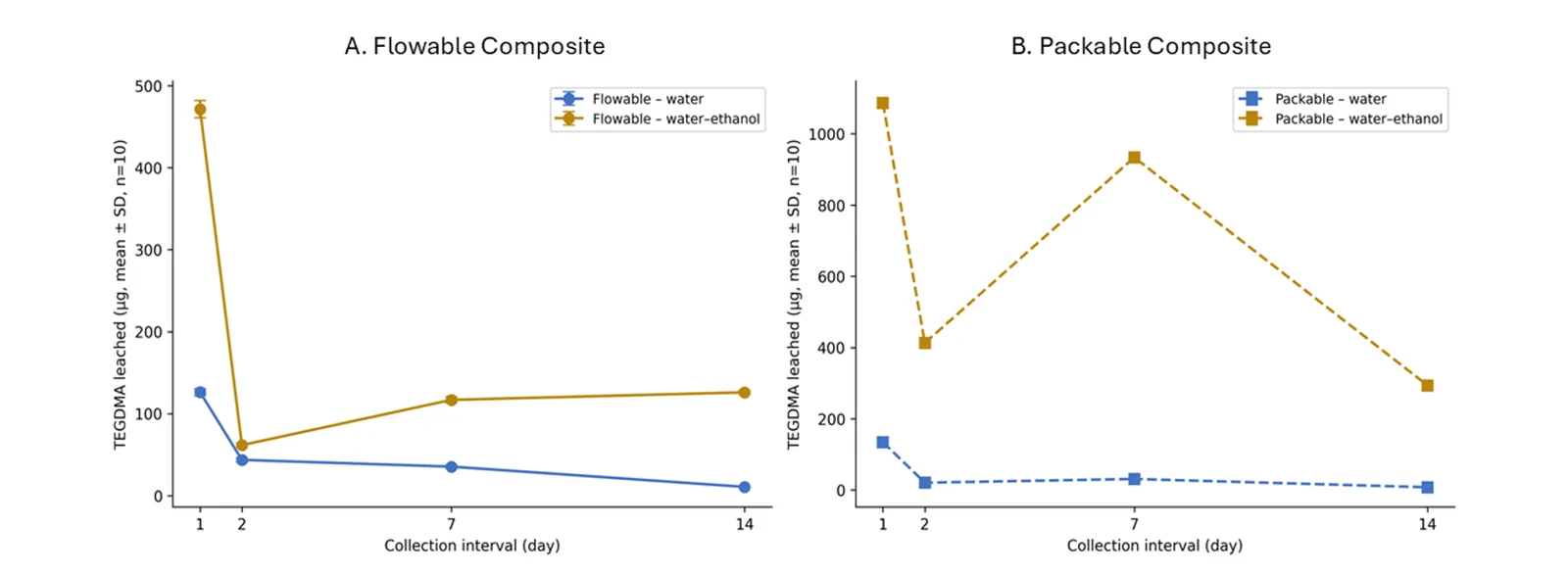
Measured TEGDMA/methacrylate leaching from specimens immersed in 1 mL of extraction medium (mean ± SD, n = 10 specimens per point, each sampled repeatedly at all four intervals) by composite, medium, and collection interval. Flowable/water-ethanol values represent triethylene glycol dimethacrylate (TEGDMA)-calibrated methacrylate equivalents. ​​​(A) Flowable composite: a simple monotonic decline under water immersion (solid blue circles) contrasted against a sharp drop and subsequent rise in a 1:1 water-ethanol medium (solid gold circles).​​​​​​​ (B) Packable composite: a distinct non-monotonic profile across both media (dashed lines, square indices), characterized by a sharp initial decrease at Day 2, followed by a significant secondary release peak at Day 7. Created by the author with Adobe Illustrator software (Adobe Inc., San Jose, CA)

Tukey HSD post-hoc comparisons were calculated separately for the two comparison families implied by the split-plot design, using the uncorrected (nominal) error degrees of freedom, consistent with standard post-hoc practice; the Greenhouse-Geisser correction above applies to the omnibus within-specimen F-tests, not to these pairwise comparisons. For the between-specimen comparison of medium within each composite, pooled across the four intervals (q = 3.81, HSD = 3.16 µg, df = 36, specimen-nested error term), water-ethanol exceeded water for both the flowable composite (193.9 vs 53.9 µg mean) and the packable composite (681.5 vs 48.3 µg mean; both p < 0.05). For the within-specimen, day-to-day comparisons (q = 3.69, HSD = 4.61 µg, df = 108, interval × specimen residual error term), every successive day-to-day change was significant in all four material × medium conditions, including the Day 2-7 rises discussed below.

Temporal pattern of leaching

The temporal pattern of relative release was not uniform across the four material × medium combinations. The flowable composite in water was the only condition to show a simple, monotonic decline across all four intervals (126.1 → 43.5 → 35.3 → 10.6 µg; each successive drop significant by Tukey HSD), consistent with a straightforward diffusion-limited depletion process. The remaining three combinations all showed a sharp initial fall from Day 1 to Day 2, followed by a significant secondary rise at Day 7, rather than a continued decline: flowable in water-ethanol fell from 471.5 to 61.5 µg TEGDMA-equivalent and then rose to 116.7 µg TEGDMA-equivalent (Day 7) and 125.9 µg TEGDMA-equivalent (Day 14); packable in water fell from 134.2 to 20.5 µg and then rose to 31.0 µg (Day 7) before falling again to 7.6 µg (Day 14); and packable in water-ethanol fell from 1086.1 to 412.7 µg and then rose sharply to 933.7 µg (Day 7) before falling again to 293.5 µg (Day 14). Every one of these day-to-day changes, including the Day 2-7 rises, was statistically significant (Tukey HSD, p < 0.05). A single-exponential, diffusion-limited decay model therefore describes only one of the four material-medium combinations examined; the other three are more consistent with an early burst release, followed by a distinct secondary release phase around Day 7.

Scanning electron microscopy findings

Before immersion, representative control specimens showed a smoothly cured surface adjacent to a fracture facet already present in the mounted specimen (Figure 4A, Figure 5A). Only minor additional porosity (isolated entrapped-air voids up to approximately 1 mm across) was noted elsewhere on the cured surface at baseline.

**Figure 4:**
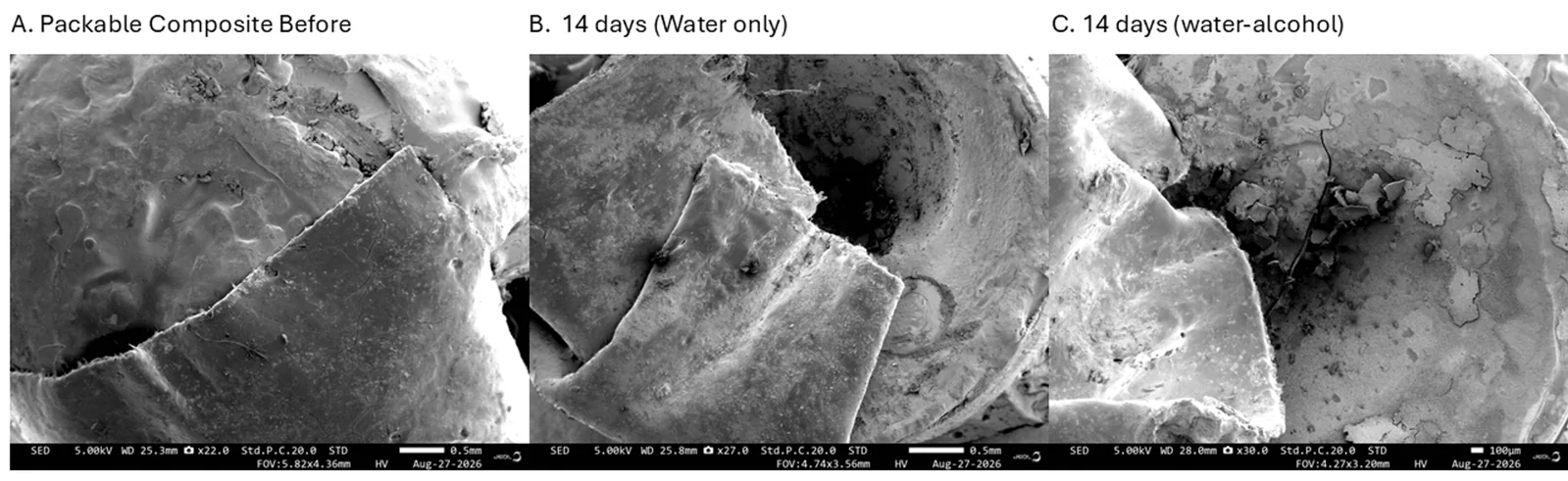
Representative scanning electron microscopy (SEM) overview of the packable composite. (A) Before immersion, showing a smoothly cured surface adjacent to a fracture facet already present in the mounted specimen. (B) After 14 days in deionised water, showing further crack propagation and a delaminated flap adjacent to an exposed internal void. (C) After 14 days in the water-ethanol (1:1) medium, showing a comparable crack and a patch of flaking, plate-like surface material. Acquisition parameters (accelerating voltage, magnification, field of view, scale bar) are embedded in each micrograph.

**Figure 5:**
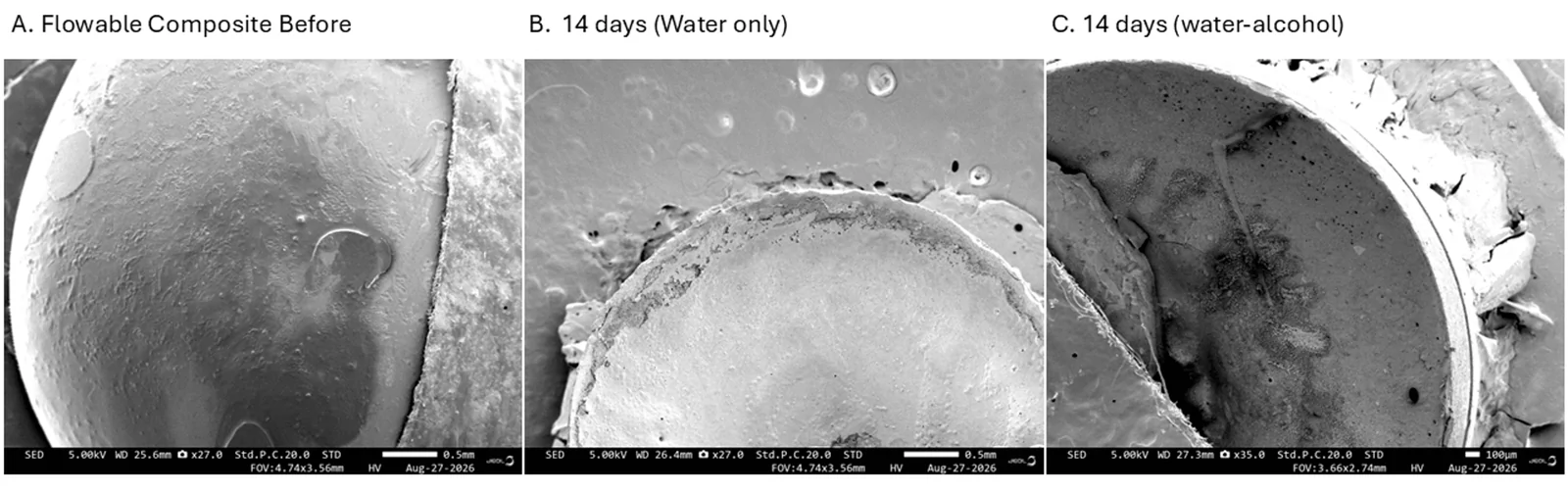
Representative scanning electron microscopy (SEM) overview of the flowable composite. (A) Before immersion, showing a smoothly cured surface adjacent to a fracture boundary already present in the mounted specimen plus isolated pre-existing fabrication voids. (B) After 14 days in deionised water, showing a fracture flap exposing larger (~0.40-150 µm) voids. (C) After 14 days in the water-ethanol (1:1) medium, showing a crack bordered by a zone of fine, dark surface stippling. Acquisition parameters are embedded in each micrograph.

After 14 days of continuous immersion in either deionised water or the 1:1 water-ethanol medium, both specimens showed clear additional disruption relative to this baseline: a fine crack propagating across the facet, and localised flaking or delamination of a thin surface layer into irregular plates immediately adjacent to it, in both media (Figures 4B-4C, Figures 5B-5C).

At the higher magnifications available for the water-ethanol-immersed specimens only, both specimens' surfaces additionally showed fine, filler-particle-scale pitting - predominantly 0.4-0.6 µm in diameter, with a smaller population extending to approximately 20-30 µm, estimated from the embedded scale bar - concentrated in patches around the crack (Figure 6). This is reported as a descriptive observation specific to the water-ethanol images examined, consistent with selective loss of surface filler particles or filler-poor surface resin at that magnification; it is not evidence that water-immersed surfaces lack comparable fine structure, which was not evaluated at a matching magnification.

**Figure 6:**
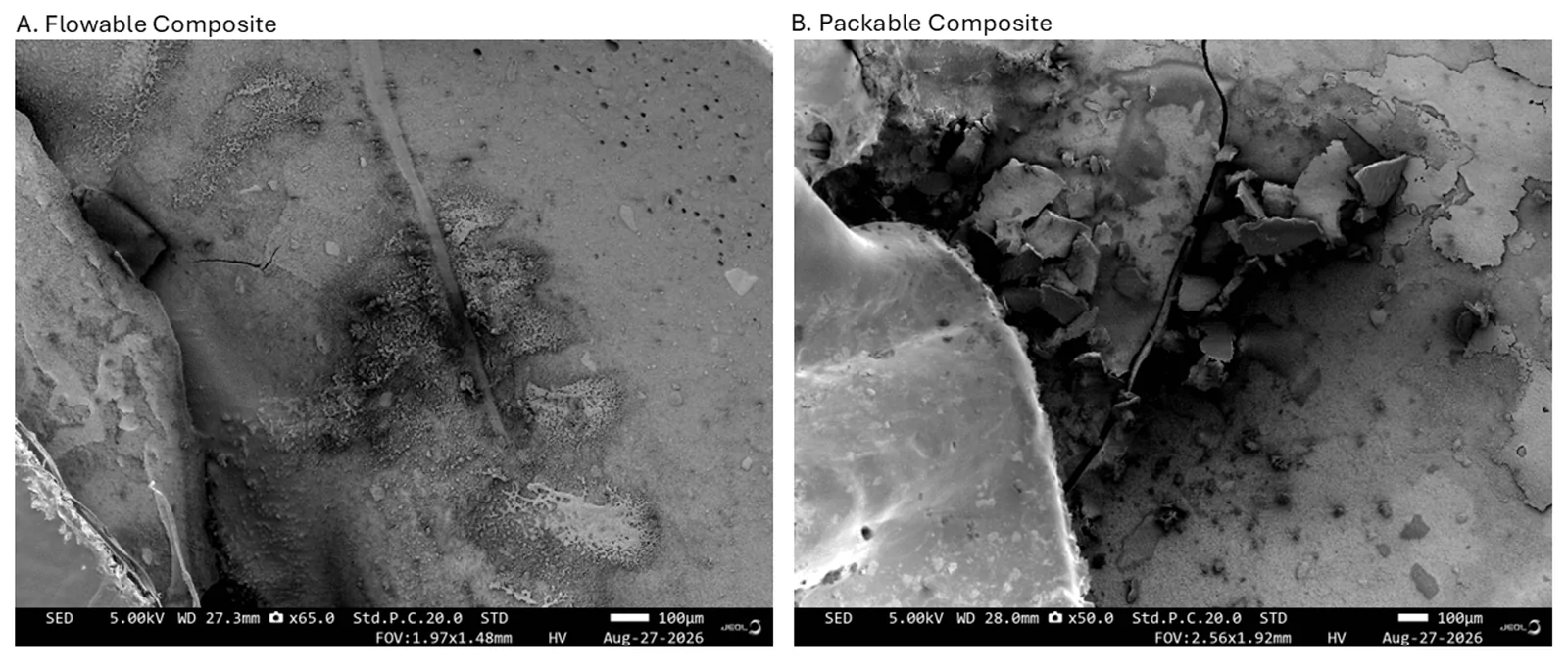
High-magnification detail of the crack-adjacent surface in the water-ethanol (1:1)-immersed specimens after 14 days of immersion. (A) Specimen A (flowable), ×65, showing a denser field of fine (~0.4-0.6 µm) pits alongside the propagating crack. Scale bars, 100 µm. (B) Specimen B (packable), ×50, showing plate-like flaking and fine surface stippling.

## Discussion

This study combined HRMS, UV-Vis spectrophotometry, and split-plot three-way ANOVA to quantify the identity, magnitude, and time course of TEGDMA relative release from a flowable and a packable composite. Of the three a priori hypotheses, the first two were confirmed: TEGDMA was the most consistently detected species, and water-ethanol produced significantly greater leaching than water overall for both composites (Tukey HSD, p < 0.05, pooled across intervals). The third - a progressive decline in leaching across successive intervals - held for only one of the four material-medium combinations; the other three showed a significant secondary rise partway through the observation period, addressed in detail below. TEGDMA relative leaching differed significantly by composite, medium, interval, and every interaction among them (all p < 0.001), and the packable composite released substantially larger absolute quantities than the flowable composite in water-ethanol. That combination of findings - significant three-way interaction, not just three significant main effects - is the central result: it means the amount of TEGDMA relative released cannot be predicted from composite type or medium alone, but depends on the specific combination of material, solvent, and time since immersion. Composite restorations number in the millions annually and are increasingly displacing amalgam in low- and middle-income settings, so this condition-specific behaviour has direct relevance to estimating real-world monomer exposure rather than being a laboratory curiosity.

The dominance of TEGDMA as a leachable species fits its physicochemical profile: as the smallest and most hydrophilic of the four monomers considered here, it has the highest diffusion coefficient within the cured network and the greatest aqueous solubility, favouring both its escape from the matrix and its detection in predominantly aqueous eluates [2,4]. Sideridou and Achilias, using HPLC in a 75% ethanol-water medium, similarly found TEGDMA and other low-molecular-weight monomers eluting more readily than the more hydrophobic Bis-GMA and Bis-EMA from comparable composites [4]. Lempel et al. reported substantial TEGDMA and UDMA elution, alongside measurable degree-of-conversion deficits, specifically in flowable bulk-fill composites [5]. The limited detection of Bis-GMA and Bis-EMA in this study likely reflects their greater lipophilicity and molecular bulk, which restrict mobility in an aqueous or semi-aqueous medium, consistent with prior work showing Bis-GMA elution is disproportionately favoured by purely organic or high-ethanol solvents rather than by water or 1:1 water-ethanol mixtures [1,4]. The higher absolute TEGDMA quantities released by Charisma Smart, relative to Fusion Flo, in the present dataset were not anticipated: flowable composites typically carry less filler and proportionally more resin matrix, which has previously been associated with greater overall monomer release [5]. A plausible explanation is that Charisma Smart uses a higher initial TEGDMA weight fraction as a diluent monomer, or has a lower degree of conversion, than Fusion Flo, neither of which was independently verified in this study; manufacturer-declared monomer ratios were not disclosed for either product, and this comparison should be read strictly as a difference between these two specific proprietary formulations rather than a generalisable trend between packable and flowable composites as categories.

The greater leaching seen in water-ethanol than in water for both composites, confirmed here by Tukey HSD, is one of the most consistently replicated findings in this literature; the raw condition means were higher in water-ethanol than in water at every one of the four intervals for both materials (Table 2), consistent with a robust overall effect of medium. Van Landuyt et al.'s meta-analysis confirmed that organic or ethanol-containing media reliably extract more resin components than purely aqueous media, attributed to ethanol's greater solvating capacity for the largely non-polar dimethacrylate backbone shared by all four candidate monomers [1]. Ethanol both plasticises the polymer network, easing diffusion of trapped monomer, and dissolves the released monomers more effectively than water alone [2]. It is not physiologically representative of resting saliva or of any single real-world exposure - typical mouth rinses contain roughly 10-22% alcohol, and dietary or rinse contact is transient and diluted by saliva, both far short of a continuous 1:1 (~50%) immersion - and is better characterised as an accelerated, aggressive extraction model than as a direct surrogate for any specific exposure. Its value lies in estimating an upper-bound ceiling for monomer release under stressed solvent conditions, deliberately exceeding realistic dietary or oral-hygiene alcohol exposure, rather than in reproducing it [11].

The temporal pattern was the most unexpected finding. Only the flowable composite in water followed the simple diffusion-limited decline generally assumed in this literature, in which the readily accessible near-surface monomer fraction is depleted early, and release then slows as diffusion from deeper within the specimen becomes rate-limiting [2,5]. The other three material-medium combinations instead showed an early burst release (Days 1-2), followed by a significant secondary rise around Day 7 before falling again or continuing to rise by Day 14; a single-exponential decay model does not fit this pattern. Because the same individual specimens were followed longitudinally across all four intervals rather than resampled from a fresh batch at each timepoint, this rise cannot be attributed to specimen-to-specimen fabrication variability and instead reflects a genuine change in release behaviour within each specimen over time. A purely ethanol-driven mechanism does not, on its own, account for the full pattern: the packable composite in water alone also showed a significant Day 2-7 rise (20.5 → 31.0 µg, Tukey HSD), and pure water is not expected to plasticise a cured dimethacrylate network as effectively or as rapidly as ethanol. Two classes of explanation remain candidates, and the present data cannot distinguish between them. First, a secondary, delayed release phase could arise from solvent-driven swelling generally - ethanol-specific plasticisation in the water-ethanol conditions, and hygroscopic water sorption and associated matrix swelling in the water-only conditions [2], a mechanism not requiring ethanol - progressively opening additional diffusion pathways beyond the initial surface-depletion phase. Second, because the rise appears at the same nominal interval (Day 7) across three of the four material-medium combinations, including conditions with otherwise very different release chemistry, a session-specific factor common to all specimens processed around that collection point (e.g., an incubation temperature fluctuation, or a systematic handling or measurement difference at that specific collection session) cannot be excluded and would, if present, affect all conditions simultaneously regardless of solvent. Longer-term elution studies using longitudinal, same-specimen sampling have reported that monomer release, while slowing, can continue for weeks to months after initial exposure [15], which is consistent with a genuine extended release process but does not itself distinguish it from a session-specific artefact. The present four-timepoint design cannot resolve which explanation is correct; denser intermediate sampling between Day 2 and Day 7, together with an independent check on incubation conditions and handling procedures at each collection session, would be needed in a follow-up study before the Day 7 rise is attributed to a genuine release mechanism rather than a shared measurement artefact.

These mechanisms, detailed in the Introduction, situate the present findings: TEGDMA's cytotoxic and genotoxic effects typically appear in the low millimolar range, roughly 2-8 mM [6,7], concentrations considered plausible at the pulp-dentine interface beneath a fresh restoration, so the quantities measured here - tens to over a thousand micrograms per specimen, depending on condition - carry biological and analytical weight, even though no cytotoxicity assay was performed on the present eluates directly. Some studies suggest using a putty-based biomaterial layer to stop percolation of monomer or any leaching substance into the dentinal tubules, therefore preventing the effect of monomer directly on the pulp [16]. A related concern attaches to Bis-GMA-derived BPA: human biomonitoring studies have documented transient salivary and urinary BPA elevations after composite placement, and dedicated LC-MS/MS release studies confirm measurable long-term BPA release from BPA-containing composites [12,13]. Bis-GMA itself was not detected here, but the consistent, medium- and material-dependent TEGDMA kinetics observed are broadly concordant with this wider evidence and support continued surveillance of resin-composite leachates, particularly where composite use is expanding fastest [1,13].

UDMA's detection specifically in the flowable composite's water-ethanol eluate, with no corresponding HRMS signal in the packable material, may reflect differences in matrix formulation between the two categories, though this qualitative species-detection finding should be read separately from the quantitative TEGDMA comparison above; it may reflect genuinely absent or lower UDMA release from the packable composite, or simply a concentration below the HRMS detection threshold.

The SEM findings corroborate physical surface disruption alongside the chemically detected monomer release, without themselves demonstrating monomer loss. The fine (4-30 µm) filler-scale pitting observed in the water-ethanol images was captured only at magnifications not applied to the water-immersed specimens, so this observation is not used here to infer a solvent-dependent difference in fine surface structure; as noted above, the SEM findings should be read as a general, qualitative record of surface disruption following immersion, not as comparative or confirmatory evidence regarding which medium produced greater structural change.

Limitations

The study has several limitations, including limited sampling intervals, qualitative HRMS/SEM assessment, and the inability of single-wavelength UV-Vis to distinguish TEGDMA from UDMA. The proprietary formulations were not independently verified for filler content, monomer composition, or degree of conversion, limiting generalisability. In vitro extraction conditions do not fully replicate the oral environment, and biological effects were not directly assessed. Despite these limitations, consistent effects of composite, medium, and time support the robustness of the findings. Future studies should incorporate denser sampling, verified material composition, and direct biological testing.

## Conclusions

Both composites released measurable residual TEGDMA, with greater leaching in water-ethanol and higher absolute release from the packable composite. Leaching showed a delayed rise around Day 7 in most conditions, highlighting the importance of longer and denser sampling. These findings may demonstrate measurable relative monomer release and warrant further investigation of potential biological systemic relevance. SEM qualitatively showed surface cracking, flaking, and fine filler-scale pitting after immersion.

## References

[1] Van Landuyt KL, Nawrot T, Geebelen B (2011). How much do resin-based dental materials release? A meta-analytical approach. Dent Mater.

[2] Ferracane JL (2006). Hygroscopic and hydrolytic effects in dental polymer networks. Dent Mater.

[3] Powers JM, Wataha JC (2004). Dental Materials: Properties and Manipulation. 9th Edition. https://books.google.com.ph/books/about/Dental_Materials.html?id=goFN49XPfZQC&redir_esc=y.

[4] Sideridou ID, Achilias DS (2005). Elution study of unreacted Bis-GMA, TEGDMA, UDMA, and Bis-EMA from light-cured dental resins and resin composites using HPLC. J Biomed Mater Res B Appl Biomater.

[5] Lempel E, Czibulya Z, Kovács B (2016). Degree of conversion and BisGMA, TEGDMA, UDMA elution from flowable bulk fill composites. Int J Mol Sci.

[6] Lovász BV, Berta G, Lempel E, Sétáló G Jr, Vecsernyés M, Szalma J (2021). TEGDMA (triethylene glycol dimethacrylate) induces both caspase-dependent and caspase-independent apoptotic pathways in pulp cells. Polymers (Basel).

[7] Galler KM, Schweikl H, Hiller KA, Cavender AC, Bolay C, D'Souza RN, Schmalz G (2011). TEGDMA reduces mineralization in dental pulp cells. J Dent Res.

[8] Schweikl H, Spagnuolo G, Schmalz G (2006). Genetic and cellular toxicology of dental resin monomers. J Dent Res.

[9] Huang FM, Kuan YH, Lee SS, Chang YC (2015). Cytotoxicity and genotoxicity of triethyleneglycol-dimethacrylate in macrophages involved in DNA damage and caspases activation. Environ Toxicol.

[10] Chang HH, Chang MC, Huang GF (2012). Effect of triethylene glycol dimethacrylate on the cytotoxicity, cyclooxygenase-2 expression and prostanoids production in human dental pulp cells. Int Endod J.

[11] Shehata M, Durner J, Eldenez A (2013). Cytotoxicity and induction of DNA double-strand breaks by components leached from dental composites in primary human gingival fibroblasts. Dent Mater.

[12] Fleisch AF, Sheffield PE, Chinn C, Edelstein BL, Landrigan PJ (2010). Bisphenol A and related compounds in dental materials. Pediatrics.

[13] Paula AB, Toste D, Marinho A (2019). Once resin composites and dental sealants release bisphenol-A, how might this affect our clinical management? A systematic review. Int J Environ Res Public Health.

[14] Tichy A, Simkova M, Vrbova R, Roubickova A, Duskova M, Bradna P (2021). Bisphenol A release from dental composites and resin-modified glass ionomers under two polymerization conditions. Polymers (Basel).

[15] Putzeys E, Nys S, Cokic SM (2019). Long-term elution of monomers from resin-based dental composites. Dent Mater.

[16] Gilhotra V, Kumar T, Mittal S, Keshav V, Maakhni E, Kesarwani D (2024). Comparative evaluation of various biomaterials as pulpotomy agents in molars with symptomatic irreversible pulpitis: a randomized single-blinded single-center control trial. J Conserv Dent Endod.

